# 85965 Biomechanical analysis of vertebral body polymethylmethacrylate cement augmentation performed using two different techniques

**DOI:** 10.1017/cts.2021.642

**Published:** 2021-03-31

**Authors:** Kabir Torres, Brandon B. Carlson, Douglas C. Burton, Jacob Birlingmair, R. Sean Jackson, Joshua T. Bunch, Terrence McIff, Stephanie Robinson

**Affiliations:** 1 University of Kansas - School of Medicine; 2 Department of Orthopedics - University of Kansas Medical Center

## Abstract

ABSTRACT IMPACT: This study will answer key questions that spine surgeons have regarding techniques used in cement augmentation of vertebral compression fractures and will ultimately advance patient care for such injuries. OBJECTIVES/GOALS: The objective of this study is to determine if a difference exists in load-bearing characteristics and load-to-fracture between injecting cement anteriorly prior to screw placement versus cement augmentation via fenestrated pedicle screws. We also expect differences in load-to-failure characteristics between different cement volumes. METHODS/STUDY POPULATION: This study will be performed in a bioengineering laboratory that has access to a Materials Testing System (MTS). Eight cadaveric specimens will be selected from our stock after pre-screening via CT for inclusion and exclusion criteria. The levels T8-L1 will be dissected from the vertebral column along with any soft tissue structures. The vertebral bodies will be potted in an epoxy mold. From each spine, there are 2 groups of three. One vertebral body from each spine will serve as an internal control, one will be augmented with cement via a cannula and then instrumented with a non-fenestrated screw and the third will be instrumented will a fenestrated screw and then augmented with cement. After appropriate curing time, repeat CT imaging will be completed. The specimens will then be loaded to failure and the results analyzed. RESULTS/ANTICIPATED RESULTS: We hypothesize that we will see a better anterior spread with the cannula/non-fenestrated screw method as compared to the fenestrated screw. The reason being is that we would expect the fenestrated screw to experience more cement extruding from the fenestration rather than being directed anteriorly. We believe a better anterior spread of the cement will lead to a greater load-bearing capacity for the vertebral body. We also believe that a difference will exist in load-to-failure testing with the two volumes being tested, though we cannot predict to what a degree this difference will be impactful as there have been few studies prior looking at this. DISCUSSION/SIGNIFICANCE OF FINDINGS: This study is significant because it will aid in determining the optimal technique to implement in the setting of vertebral compression fractures. This will lead to improved patient care as well as a greater understanding of the instrumentation used in such procedures. The results will lay the groundwork for future research on this procedure.

